# Health Worker Perspectives on Barriers and Facilitators of Assisted Partner Notification for HIV for Refugees and Ugandan Nationals: A Mixed Methods Study in West Nile Uganda

**DOI:** 10.1007/s10461-021-03265-1

**Published:** 2021-04-21

**Authors:** Robin E. Klabbers, Timothy R. Muwonge, Emmanuel Ayikobua, Diego Izizinga, Ingrid V. Bassett, Andrew Kambugu, Alexander C. Tsai, Miranda Ravicz, Gonnie Klabbers, Kelli N. O’Laughlin

**Affiliations:** 1 Faculty of Health, Medicine, and Life Sciences, Maastricht University, Maastricht, the Netherlands; 2 Infectious Diseases Institute, College of Health Sciences, Makerere University, Kampala, Uganda; 3 Department of Medicine, Massachusetts General Hospital, Boston, MA USA; 4 Center for Global Health and Mongan Institute, Massachusetts General Hospital, Boston, MA USA; 5 Department of Internal Medicine and Pediatrics, Massachusetts General Hospital, Boston, MA USA; 6 Departments of Emergency Medicine and Global Health, University of Washington, Seattle, WA USA; 7 Harvard Medical School, Boston, MA USA; 8 Mbarara University of Science and Technology, Mbarara, Uganda; 9 Department of Health, Ethics and Society, Faculty of Health, Medicine, and Life Sciences, Maastricht University, Maastricht, the Netherlands

**Keywords:** HIV, Partner notification, Uganda, Mixed methods, Refugee

## Abstract

Assisted partner notification (APN) is recommended by the World Health Organization to notify sexual partners of HIV exposure. Since 2018, APN has been offered in Uganda to Ugandan nationals and refugees. Distinct challenges faced by individuals in refugee settlements may influence APN utilization and effectiveness. To explore APN barriers and facilitators, we extracted index client and sexual partner data from APN registers at 11 health centers providing care to refugees and Ugandan nationals in West Nile Uganda and conducted qualitative interviews with health workers (N = 32). Since APN started, 882 index clients participated in APN identifying 1126 sexual partners. Following notification, 95% (1025/1126) of partners tested for HIV; 22% (230/1025) were diagnosed with HIV with 14% (139/1025) of tested partners newly diagnosed. Fear of stigma and disclosure-related violence limit APN utilization and effectiveness. Prospective research involving index clients and sexual partners is needed to facilitate safe APN optimization in refugee settlements.

## Introduction

Despite the expansion of HIV testing and counseling services and the improvement in HIV prognosis that followed the introduction of antiretroviral therapy (ART) [1, 2], 38% of people living with HIV (PLHIV) worldwide do not access treatment, many present for initial treatment at late stages of disease [3, 4], and approximately 770,000 people die from AIDS-related illnesses each year [5]. A major factor contributing to delays in treatment initiation and increased HIV transmission remains the high percentage of PLHIV who are unaware of their serostatus [5]. Of those who know their HIV serostatus, many refrain from disclosing to their sexual partners [6–9]. Assisted partner notification (APN) for HIV is a public health strategy in which trained providers assist PLHIV in notifying their sexual partners of potential HIV exposure so they can be tested and linked to care [10, 11]. Historically, partner notification services have been used to identify high-risk individuals [12] in public health programs for sexually transmitted diseases such as syphilis and gonorrhea [13, 14]. In 2016, the World Health Organization (WHO) recommended APN implementation as routine part of a comprehensive package of HIV testing and care [10, 11]. Since that time, APN has been implemented in a multitude of settings including sub-Saharan Africa (SSA) where it has been shown to be cost-effective [15–18] and to increase uptake and yield of HIV testing [10, 19–25].

Following a successful APN pilot study that detected an HIV prevalence of 38% among participating partners, APN services were expanded across Uganda between 2017 and 2019 by the Ministry of Health (MoH) [26]. In Uganda, APN is offered to all newly diagnosed “index clients” aged 15 years and older who are identified through voluntary counselling and testing (VCT), provider-initiated counseling and testing (PICT) and prevention of mother-to-child transmission (PMTCT). APN participation is offered as well to all index clients previously enrolled in care who are determined to be at increased risk of transmitting disease (e.g., index clients not yet initiated on ART, index clients on ART who are not virally suppressed, and index clients with a new sexual partner or a sexually transmitted disease) [26].

The refugee population in Uganda, which currently numbers over 1.4 million [27], is offered the same APN services as Ugandan nationals at health centers in refugee settlements. Refugees form a unique group with distinct needs [28]. The hardships refugees experience in the face of prolonged displacement, multiple traumas, disruption of social networks, limited livelihood opportunities and difficulties meeting basic needs increase their vulnerability to HIV and limit their opportunities to engage in HIV care [28–37]. While APN has been shown to be highly effective in yielding new HIV diagnoses in care settings across SSA including Uganda, little is known about program utilization and effectiveness in the refugee settlement context or about how barriers and facilitators of participation in this context compare to regular care settings.

We conducted a mixed methods study at health centers in West Nile Uganda where APN services were being offered to refugees and Ugandan nationals to explore barriers and facilitators of APN and to gain insight into APN utilization and effectiveness in refugee settlements.

## Methods

### Study Setting

This study was conducted in West Nile Uganda, a region in northwestern Uganda that is bordered by the Democratic Republic of the Congo (DRC) and the Republic of South Sudan. In January 2019, West Nile hosted almost 60% of the country’s 1.2 million refugees living in refugee settlements [38]. South Sudanese refugees (794,387) form the main constituents of this population followed by refugees from the DRC (319,461) and Burundi (35,467) [38]. In West Nile, there is an adult HIV prevalence of 3.1% [39]. The prevalence among the refugee population is unknown, but previous studies in Uganda and SSA have found a lower HIV prevalence among refugees compared to host populations [37, 40]. The current study took place at 11 health centers located within or in the proximity of refugee settlements in the West Nile districts of Adjumani, Arua, Moyo and Yumbe that were offering HIV testing and counseling services to refugees and Ugandan nationals in January 2019. The proportion of refugees and Ugandan nationals varied across health centers.

### APN in Uganda

**Fig. 1:**
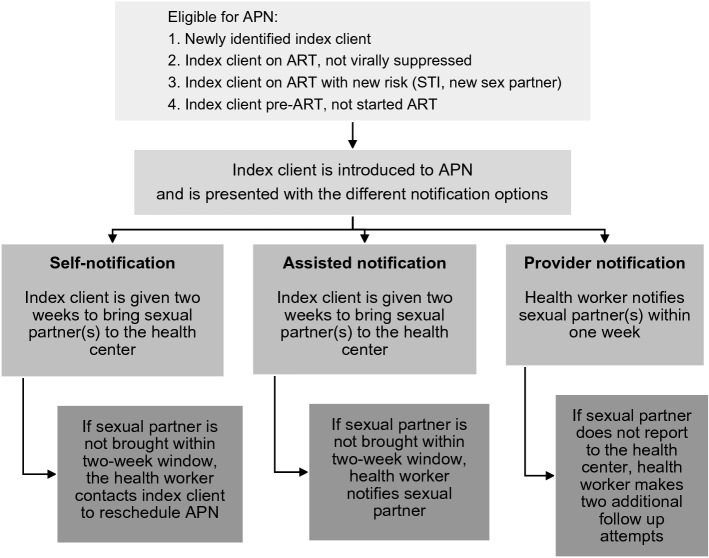
The APN notification process as defined by the Ugandan MoH

APN services were first introduced in West Nile as part of HIV testing and care in 2017 and coverage was expanded across the region throughout 2018 and 2019 [26]. As part of APN, index clients are asked to list their sexual partners and select one of the following three APN options to notify each partner of possible HIV exposure: *self-notification, assisted notification* or *provider notification* (Fig. 1, The APN notification process as defined by the Ugandan MoH) [26]. In *self-notification*, the index client notifies their sexual partner(s) and brings the partner(s) to the health center for testing (i.e. passive referral in WHO guidelines [11]). In *provider notification,* the health worker notifies the sexual partner(s) without revealing the identity of the index client and encourages the sexual partner(s) to report to the health center for testing (i.e. provider referral [11]). *Assisted notification* refers to a combination of the other two modalities in which the index client is given a two-week window to notify their sexual partner(s) after which, if unsuccessful, the health worker notifies the sexual partner(s) (i.e. contract referral [11]).

### Data Collection

Quantitative and qualitative data were collected concurrently during a single visit to each participating health center in July 2019.

#### Quantitative Data: APN and HIV Register Data Extraction

Data routinely collected and reported by clinic staff as part of HIV testing and care were de-identified and extracted from written HIV and APN registers for all index clients who participated in APN and the sexual partners they identified from the time of APN initiation at each health center (earliest December 2017, latest January 2019) until the time of this study (July 2019). Collected data included index client and sexual partner sociodemographic information (e.g. age, sex, marital status, refugee or Ugandan national status), information regarding the chosen APN notification option for each sexual partner, success of partner referral, and HIV test outcomes. A secure electronic REDCap database was used to store de-identified APN and HIV register data.

#### Qualitative Data: Interviews with Health Workers Involved in HIV Testing and Care

Anticipating we would need 20–40 interviews to reach data saturation [41–44], two to three health workers were identified at each of the 11 health centers and approached for study participation. Inclusion criteria were involvement in HIV testing and care, age over 18 years, and English fluency. Based on these criteria, no health workers were excluded. Following written informed consent, demographic data were collected for each interview participant including sex, age, years of work experience and role in APN. A semi-structured interview guide informed by Weinstein et al.’s Precaution Adoption Process Model (PAPM) [45–47] was used to interview study participants. For the interview guide, as has previously been described by Plotkin et al. [20], the PAPM was adapted to examine the different cognitive stages that sexual partners pass through when deciding to get tested for HIV following notification of possible exposure by index clients, allowing for the identification of factors influencing the APN process. Interview participants were asked about their impression of APN program effectiveness and their perspectives on barriers and facilitators of APN as well as recommendations on how the APN program could be made safer and more effective. As themes emerged from the interviews, the interview guide was iteratively refined. Interviews were conducted in private rooms at the health center. Participants received compensation for their time (20,000 UGX, equivalent to approximately 5.40 USD or 4.90 EUR). All participants gave written permission to audio-record their interviews. De-identified audio files and interview transcripts as well as the sociodemographic details of interview participants were stored in an electronic REDCap database.

### Data Analysis

#### Analysis of Quantitative Data

Data were exported from the REDCap database and analyzed using IBM SPSS Statistics (version 25) [48]. Simple frequency tables were used for descriptive analysis of index clients participating in APN, the number of identified sexual partners, chosen APN notification options, tested partners and HIV testing outcomes. Cross-tabulations with Chi-square analysis were used to determine whether choice of notification option differed significantly between refugees and Ugandan nationals and whether there were significant differences in success of partner referral for the different notification options. A Mann–Whitney U test was used to compare the median number of sexual partners identified per index client for refugees and Ugandan nationals. A p value < 0.05 was considered statistically significant.

#### Analysis of Qualitative Data

Qualitative data were analyzed using inductive thematic analysis [49]. First, a sample of five interview transcripts were read carefully by three researchers independently (REK, TRM, KNO) to identify data relevant to the research topic. Open coding was applied to these sections to identify and describe distinct ideas and themes, followed by the organization of themes into categories and subcategories to create an analytic coding framework. The resulting framework was discussed among the three researchers until a consensus was reached. Transcripts were reviewed a second time to evaluate exhaustiveness and clarity of themes and categories and the framework was adjusted accordingly. The final coding framework was then applied to the remaining transcripts by one researcher (REK).

Following independent analysis, quantitative and qualitative results were integrated and interpreted together allowing for corroboration and contextualization of findings [50].

## Results

### Quantitative Results: APN and HIV Register Data

#### Program Utilization

Since APN services were initiated through the date of data extraction, December 2017 through July 2019, a total of 882 index clients voluntarily participated in APN at the 11 health centers included in this study. Index clients were predominantly female (58%) and had an average age of 35 years (16–76 years, standard deviation [SD] 9.46 years). Of the index clients participating in APN, 418 index clients (47%) were refugees and 360 index clients (41%) were Ugandan nationals; for 104 index clients (12%), no information was recorded regarding refugee or Ugandan national status. Each index client identified an average of 1.3 sexual partners (modal number 1, range 1–6), corresponding with a total of 1126 listed sexual partners. The median number of sexual partners identified by Ugandan national index clients was 1 (interquartile range [IQR] 1–2), and the median number of sexual partners identified by refugee index clients was 1 (IQR 1–1) (Mann–Whitney U test for comparison, U [N_Refugee_ = 418, N_National_ = 360] = 61185.5; z = − 5.99, p < 0.001). The majority of sexual partners were male (54%) and had an average age of 34 years (16–68 years, SD 9.04) (Table 1).

**Table 1:** Sociodemographic characteristics of index clients and sexual partners

	Index clients (N = 882)	Sexual partners (N = 1126)
	Number (%)	Number (%)
**Sex**		
Male	366 (41)	604 (54)
Female	509 (58)	521 (46)
Data missing	7 (1)	1 (< 1)
**Age**		
Mean (range, SD)	35 (16–76, SD 9.46)	34 (16–68, SD 9.04)
15–18 years	13 (1)	9 (1)
19–24 years	91 (10)	108 (10)
25+ years	753 (85)	986 (88)
Data missing	25 (3)	23 (2)
**Refugee/national status**		
Refugee	418 (47)	
National	360 (41)	
Data missing	104 (12)	
**Partners listed by**		
Refugee index clients		481 (43)
National index clients		516 (46)
Data missing		129 (11)
**Marital status**		
Married/cohabitating	655 (74)	
Never married	69 (8)	
Separated/divorced	96 (11)	
Widowed	34 (4)	
Data missing	28 (3)	
**Index client type**		
Newly identified	450 (51)	
On ART not virally suppressed	152 (17)	
On ART with new risk (STI, new partner)	134 (15)	
Pre-ART, not started ART	8 (1)	
Data missing	138 (16)	

#### Choice of APN Notification Option

**Fig. 2:**
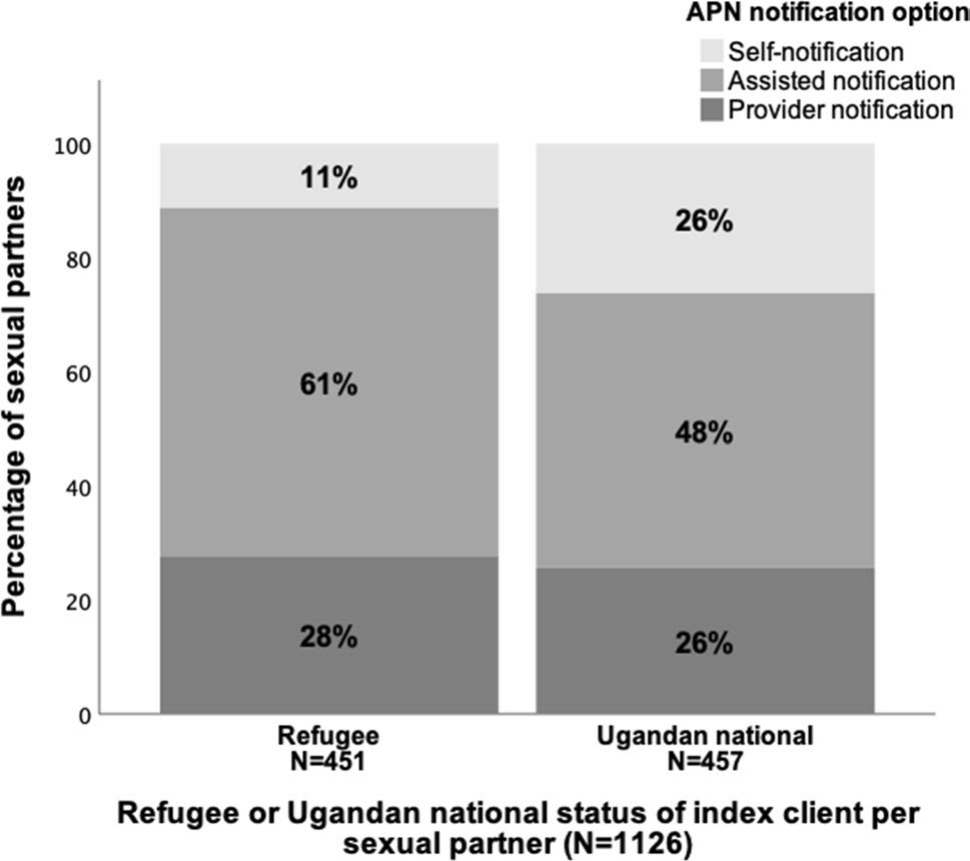
Distribution of APN notification option choice for each sexual partner by refugee and Ugandan national status of the index client. *Data on refugee or Ugandan national status of the index client or APN notification option missing for 218/1126 sexual partners

The notification option most commonly chosen by index clients to notify their sexual partners of possible HIV exposure was *assisted notification* (*assisted notification* 51%, *provider notification* 31%, *self-notification* 18%) with the distribution of notification options varying across the different health centers. In 81% of the cases where *self-notification* was selected (149/185 cases), APN registers reflected that sexual partners received a phone call or a home visit from the health worker suggesting that index clients did not succeed in notifying their sexual partner(s) themselves, and assistance by a health worker was later implemented. Thus, although recorded as *self-notification* in the APN registers, ultimately these cases defaulted to *assisted notification*. For this study, these cases were analyzed according to how they were recorded in APN registers—i.e. as *self-notification*. Refugee and Ugandan national index clients displayed different notification option preferences. Chi-square analysis comparing the distribution of the three notification options for refugee and Ugandan national index clients revealed a statistically significant difference between the two groups with Ugandan nationals opting for *self-notification* in a greater percentage of cases than refugees (26% and 11% respectively, p < 0.001) (Fig. 2, Distribution of APN notification option choice for each sexual partner by refugee and Ugandan national status of the index client).

#### Testing of Sexual Partners and HIV Test Outcomes

Following notification of possible HIV exposure through APN, 91% (1025/1126) of sexual partners reported to the health center and consented to HIV testing. Of the three notification options, *self-notification* was the least effective in bringing sexual partners for HIV testing with 87% (161/185) of sexual partners tested versus 96% (498/521) and 91% (290/319) for *assisted* and *provider notification* respectively (p < 0.001). Of the 1025 partners who were tested, 22% (230/1025) were diagnosed with HIV. Of these sexual partners diagnosed with HIV, 60% (139/230) were newly identified index clients and 36% (83/230) were index clients who were already enrolled in care, corresponding with a new HIV diagnosis yield of 14% (139/1025) for all tested sexual partners.

### Qualitative Results: Health Worker Perspectives

Qualitative interviews were conducted with 32 health workers holding positions related to HIV testing and care delivery. Interview participants had a mean age of 32 years, were predominantly male (53%) and had a mean work experience of 5 years (Table 2).

**Table 2:** Sociodemographic characteristics of health worker participants

Interview participant demographics (N = 32)	
**Gender N (%)**	
Male	17 (53)
Female	15 (47)
**Mean age in years**	32 (20–48, SD 7.52)
**Work experience**	
Mean total work experience in years	5 (1–23, SD 4.50)
Mean experience at health center in years	3 (0.8–8, SD 1.95)
**Position at health center N**	
Counselor	9
ART clinic in charge (nurse in charge)	6
Linkage and retention facilitator	6
Midwife	3
Clinical officer	2
Nurse	2
Health center in charge	1
Expert client	1
Volunteer	1
Community Drug Distribution Point project assistant	1

#### Impression of the APN Program

Health workers spoke positively about APN, recognizing that the approach had enabled the identification of HIV-exposed individuals, including sexual partners living further away from the health center and specific groups with limited health-seeking behavior such as men.

*APN Effectiveness* Health workers reported that APN yielded new HIV diagnoses and believed the program did so more effectively than previous approaches such as home-based testing. Additionally, health workers valued the importance of sexual partners learning their seronegative status through APN as it enabled counseling on HIV prevention.

When speaking about APN effectiveness, health workers made a distinction in effectiveness between the three different notification options. Little consensus was found however, regarding the definitions of the three notification options despite their clear outline in APN guidelines (Fig. 1, The APN notification process as defined by the Ugandan MoH) [26]. When talking about *assisted notification*, most interview participants referred to any form of notification in which assistance was provided by a health worker, as opposed to *self-notification* in which index clients notify their sexual partner(s) themselves. The distinction between *assisted notification* and *provider notification* was unclear for many health workers and very few interview participants were able to name the two-week window described in the guidelines that index clients are given in *assisted notification* to bring their partners to the health center before health worker disclosure is implemented, which differentiates this option from *provider notification* [26]. As a result, health workers spoke almost exclusively about *self-notification* and *assisted notification* in the interviews and rarely mentioned *provider notification*.

*Assisted notification* was reported to be the most effective method to bring sexual partners to the health center for HIV testing. Health workers explained that when index clients opt for *self-notification* it usually takes a long time for them to gather the courage to disclose their status; it was estimated that for most index clients who choose *self-notification*, a switch to an alternative form of disclosure proves necessary. The strength of *assisted notification* was considered that control lies with the health worker, thereby preventing significant delays in reaching sexual partners. *Assisted notification* was also said to allow for counseling on the importance of testing even if feeling healthy, something index clients were are not always able to successfully convey to their sexual partners in *self-notification*.

Health workers reported that the most effective notification strategy, *assisted notification*, was also the notification option most commonly selected by index clients. Notification option preference was thought to be the culmination of multiple factors and was considered to be strongly motivated by a preference for confidentiality and fear of negative consequences such as violence, accusations of promiscuity or infidelity by sexual partners, and stigma.

Health worker (54-05-2-002), male: “They fear their partners, so they feel it is better if they can give this information to the health worker, and then it is the health worker who uses the knowledge of their training to convince the partner. It is better. Then they also feel safer.”

Fear was also identified by the health workers as a reason why some index clients prefer *self-notification*. As described by health workers, some index clients believe that disclosing to sexual partners themselves prevents third parties from becoming involved and aware of their status. A visit from the health worker in the community—as would take place in the case of *assisted notification* (after the initial two-week self-notification window) or *provider notification*—could raise questions and lead to stigmatization, ostracization or abandonment by family and community members.

#### Barriers to APN

**Fig. 3:**
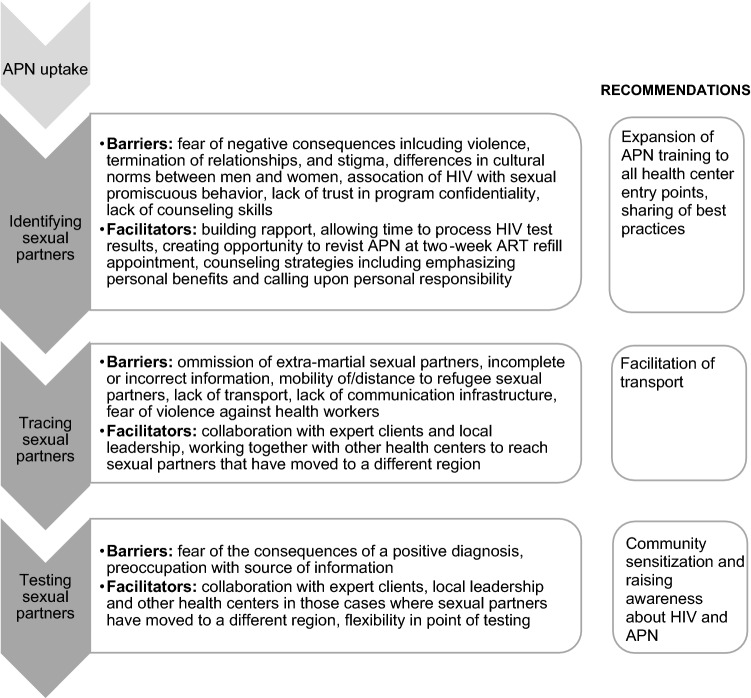
Health worker perspectives on barriers and facilitators of APN and corresponding recommendations to enhance APN safety and effectiveness

Although health workers considered APN to be an effective strategy to identify sexual partners, they explained that many barriers impede program effectiveness. Barriers exist at all program levels including the identification of sexual partners by index clients, tracing of sexual partners, and testing of sexual partners after they have been notified of possible HIV exposure (Fig. 3, Health worker perspectives on barriers and facilitators of APN and corresponding recommendations to enhance APN safety and effectiveness). Some barriers were reported to be specific to the refugee population, whereas others were said to be applicable for refugees and Ugandan nationals alike. Reported barriers are applicable to index clients and sexual partners from both populations unless otherwise specified.

*Barriers to Identifying Sexual Partners* One of the major barriers to identifying sexual partners reported by health workers was the fear from index clients that listing their sexual partners and disclosing possible HIV exposure would lead to accusations of infidelity, violence, termination of relationships, and stigma by the community. This was noted particularly for women given differences in the number of sexual partners considered culturally acceptable for men compared to women.

Health worker 54-10-2-002, female: “Because in our setting, women are supposed to have one husband, it is men who have very many women. . . . So, it is making women to fear disclosing. . . . They are afraid of violence of course, from the husbands, even the relatives of the husband. Because they will say, ‘You are cheating me, you are’ and even divorce.”

Health worker 54-05-2-002, male: “First of all, it is stigma. Yes. They feel that when they disclose their status to their partner, the partner is going to spread the news to other people whereby they are going to start talking ill about him or her. So, they feel stigmatized.”

Health workers explained that in this setting, a positive HIV status is considered strongly linked to promiscuous sexual behaviour and can tarnish one’s reputation in the community.

Health worker 54-02-2-001, male: “It is hard because you know, in our locality here, it is like when you get HIV it is like you are a sex worker. . . . They may think you can get this virus through sex only. That is why they get a fear.”

Health worker 54-06-2-001, female: “Commonly, they will think someone is immoral, like sexually over-involved. . . . So, if you have HIV, directly translates that you have been involving in unsafe sex which is not a good representation. . . . especially women, they would like to marry more. . . . HIV, it will destroy now their market if she is interested in marrying again.”

Health workers reported that consequently, index clients who do agree to identify their sexual partners frequently omit casual partners and extra-marital relationships.

Health worker 54-03-2-001, male: “Usually, many of these clients, they are telling their partners whom they have got married to, but the outside one whom they have not yet married to, they are not mentioning this.”

While health workers reported that HIV is highly stigmatized in both refugee and Ugandan national communities, they explained that the negative consequences of HIV disclosure differ in severity for these two groups, and as a result, may act as a stronger deterrent to listing sexual partners for refugees than for Ugandan nationals.

Health worker 54-05-2-002, male: “Like for the refugees, in their culture, if somebody is HIV positive . . . we are supposed to do away with that person—either by sending him or her far away, or else you will kill the person . . . killing somebody, sending the person far away, for us [Ugandans] it is not there . . . targeting or stigmatizing [of] the person that is the greatest challenge we are facing here with the nationals.”

Cultural differences, exposure to interpersonal violence while fleeing to Uganda, and lack of HIV awareness in their countries of origin were suggested by health workers to contribute to the more severe reactions to HIV disclosure seen in refugees compared to Ugandan nationals.

Health worker 54-07-2-002, male: “You know these refugees . . . they are traumatized. They are really difficult. Have much [violence] in spite the continuous counseling that we have always offered for them.”

Health worker 54-06-2-001, female: “For us in Uganda we are now well conversed with HIV and super comfortable with result [receiving a positive diagnosis]. Yes, they [Ugandans] have personal reactions, but no violence. But up there [Sudan], . . . to them [refugees] it is a bit more fresh, it is hard for them to comprehend, so they may think you are terrifying them, you are lying . . . disclosing to them is not as easy as disclosing to nationals. You take extra care and time and massaging to bring them to . . . the point compared to the nationals . . . the information on HIV in Sudan is still limited. . . . There is still a gap, we should say maybe HIV stigma is higher there than here. So, a positive result is too bad news. Here it is just bad news.”

Health workers explained that mistrust in the confidentiality of APN participation contributes to fear of listing sexual partners. This was said to be especially the case for refugee index clients for whom the APN process requires involvement of third parties such as interpreters to bridge language barriers.

Health worker 54-03-2-002, female: “For the nationals, I speak the language, there is no problem in that. . . . The issue comes with the refugees, . . . because of language barrier it will force you to get somebody to translate. Then the issue of translation comes. At times these people [the refugees] they don't like [do not trust] the translator. There they may not disclose for you fully. You may not even get the appropriate what? Information that you wanted from this person.”

The close housing proximity in the refugee settlement also limits confidentiality of APN and discourages refugee index clients from listing their partners for tracing in the community.

Health worker 54-07-2-001, male: “In refugees, we find that they . . . are highly populated . . . confidentiality in the community is not good, compared to the national where you find they are sparsely populated. [The refugee community] might see why have the health workers come here? Why have they called this man? . . . when you are going for that assisted partner notification.”

Refugee index clients who expect that tracing their sexual partners will not be feasible due to the large distances that would need to be traveled, sometimes elect not to mention sexual partners residing far away to the health worker.

Health worker 54-04-2-001, female: “Let me talk of the refugees, he is [living] in Uganda [but] maybe he works in South Sudan. He goes there, stays there for maybe one year, comes here once a year . . . the sexual partners, they can be very many . . . he cannot possibly bring them from there to here for testing, so he says no I don't have any other women, this is my wife.”

Interview participants reported that challenges in identifying sexual partners are compounded by a general lack of health worker training on counseling strategies to convince index clients to disclose their sexual partners. Most interviewed health workers stated that they had not received formal APN training. In most cases, only one or two health workers from each facility had been selected to take part in a 2–5-day APN training hosted in the region. Interviewees reported gaining knowledge on APN through alternative channels such as by learning while working, learning from colleagues who had received APN training and by acquiring skills during continuing medical education sessions offered by implementing partners.

*Barriers to Sexual Partner Tracing* In *assisted* and *provider notification*, information provided by index clients is used by health workers to trace sexual partners in the community and notify them of possible HIV exposure. Locating these sexual partners can be challenging as index clients often provide incomplete or incorrect information. Health workers suspected that sometimes this is done intentionally and other times the index clients simply do not have complete information about the sexual partner.

Health worker 54-04-2-001, female: “So some of these . . . partners they are having, is just a sexual partners. So how are you going to bring her? . . . These one-day sexual relationships mostly are sexual workers. So, you even don't know them, maybe you got this person at night, just on the street. . . . you don't have any information about her.”

Especially for refugee index clients whose husbands are away in South Sudan, information on how and where to contact sexual partners is not always available.

Health worker 54-03-2-003, female: “On the side of refugees, they say most of their husbands . . . they are for army. They are there [South Sudan] for fighting. But few have their contact, the husband contact.”

Following up on index clients and reaching sexual partners belonging to the refugee population in the community is often complicated by the high degree of mobility among refugees.

Health worker 54-08-2-003, female: “These refugees the challenge with them again is the movement—they are not in one place. When they know that they are positive here [when the community becomes aware of their HIV status] . . . they will leave.”

Health worker 54-05-2-001, male: “For refugees those we are learning that the partner may be . . . moving around from one camp to another. So . . . it is very hard to get the partner.”

Health worker 54-01-2-002, female: “Nationals . . . they are not up and down, they are not moving, like the one the refugees. But the refugees like in July you will be here, in August you will go to Sudan. They are just moving like that.”

Many sexual partners are not living in the refugee settlement and some may have even left Uganda and returned to their home country.

Interview participants described how in the case of Ugandan index clients, large distances to sexual partners could sometimes be bridged by coordinating with other health centers in Uganda. Health workers explained that for refugees with sexual partners in Sudan, however, this was not an option due to the lack of health infrastructure in Sudan.

Health worker 54-08-2-003, female: “The biggest challenge here with our settlement, like with the refugees, is the movement. Because you test one client here who say, ‘my partner is [in] Sudan’. How I am going to do that? It is hard. We usually tell . . . the woman [the index client], in case this man comes here, you bring this man to the facility. . . . We cannot trace with the health centers in Sudan. They are not coordinated. Maybe within Uganda, if they are in another district, we can call we have the contacts, we have all the contacts within here. . . . [But] the refugees they fear. They don't bring. They will even say this man has not come when the man has come and has gone back or he's around.”

Practical challenges of transport and communication further complicate partner notification. There are no vehicles available for carrying out APN services and health workers have to rely on public transportation such as boda-bodas (local motorcycle taxis) to trace sexual partners. This transportation frequently has to be personally financed by health workers who can be reimbursed later.

Health worker 54-03-2-003, female: “For me, the biggest challenge in APN, it is about first of all, it is transport, though they give transport [facilitation]. But they give after you have done the activity. Yeah, so at times you have to go and do APN when there is no money, and like those who come from far, when a long distance, we are no longer reaching there because of the transport.”

The majority of people living in the refugee settlements do not have constant access to a personal mobile phone and consequently health workers have to locate sexual partners using directions provided by index clients. Even when people do own a personal phone, there may still be barriers such as signal strength, phone credit and lack of charging facilities. In this context, tracing sexual partners becomes a process associated with many practical and logistical challenges.

Health worker 54-06-2-001, female: “Some of the challenges like in communication. Most of the clients are to be followed-up up to where they are, you reach home, they have gone to buy salt. Where they went to buy salt, they tell they went to fetch water. So that kind of things. Actually, most of them do not have phones, even in our files, so you follow them personally.”

Violence was considered another barrier to tracing sexual partners. Participants described cases in which health workers were met with hostility from the refugee community while carrying out APN activities. Violence prevents health workers from following up on certain cases in the community and makes it dangerous to carry out APN duties alone.

Health worker 54-05-2-002, male: “For the refugees it is very difficult. Like my colleague last time tested a refugee positive and up to today . . . this refugee is still in denial that she is having HIV. And she doesn't want us to follow her, so . . . for self-protection, we have also declined to go and follow on her. . . . The refugees like the Dinka [and] the Nuer, they are always very aggressive. If somebody says no and you still insist, they can harm you. A colleague it happened one time. . . . they were like chasing her with even a Panga knife. . . . they do not want themselves to be exposed to other people, because we are health workers, people within the communities know that, so, they feel that once we started following her, people are going to get concerned.”

Health worker 54-08-2-003, female: “[W]hen we are following . . . we got challenges in the community. . . . we are not allowed to go individual. At least a lady and a man. You cannot go as a lady alone. You cannot. It is not safe. Like in such scenario you will be harmed.”

*Barriers to HIV Testing of Sexual Partners After Notification* Interview participants reported that even when health workers can successfully trace and notify sexual partners of possible exposure, not all sexual partners come to the facility for HIV testing. Some partners refuse or delay HIV testing because they fear receiving a positive HIV test result and the consequences associated with a new HIV diagnosis.

Health worker 54-11-2-001, female: “They [sexual partners] are fearing . . . [they think] if I will be found positive, I may faint, because I'll not want to . . . have HIV. . . . am I going to use the drug [ART] for life? For the whole of my life? That is now the fear.”

Health worker 54-08-2-003, female: “[They are afrBuilding rapport was one of the keyaid] of knowing that they are positive. . . . They are going to take drugs. . . . They don't mind much about what the drug will do. They know that the drug helps them. But what they think is what people will say about them. Their perception about what people will say about them is too much.”

Other barriers to testing sexual partners include sexual partners’ preoccupation with being identified through APN when health workers notify them of possible exposure to HIV. Many sexual partners are more focused on figuring out why they are being singled out instead of learning about the possible HIV exposure and addressing the risk through HIV testing.

Health worker 54-08-2-002, male: “Some of them when you try to call them, they [sexual partners] . . . wanted you to tell why and who [gave their contact information], which is a bit tricky, which we don't do if it is not allowed by the partner [index client].”

Health worker 54-09-2-002, male: “They normally ask questions, ‘Why are you looking for me? So, what is the problem?’ Others at first, they get worried, because from nowhere you have sighted where he is, and you bump in and say, ‘I am so and so, and come for these services.’”

#### Facilitators of APN

To overcome the various challenges impeding APN implementation, health workers employ several strategies that facilitate the different components of APN (Fig. 3, Health worker perspectives on barriers and facilitators of APN and corresponding recommendations to enhance APN safety and effectiveness).

*Building Rapport to Encourage Listing of Sexual Partners* Building rapport was one of the key factors identified for promoting the listing of sexual partners by index clients. Health workers explained that because of the stigma that still surrounds HIV in this setting, an atmosphere of trust and friendship needs to be created over the course of several clinic appointments before index clients feel comfortable enough to disclose their partners.

Health worker 54-10-2-001, female: “You know, counselling is not easy. We first make the person friendly what what, maybe a week, two times. . . . Then you ask [about APN participation], after getting used to the person. The person will be a friend for you.”

Health worker 54-02-2-001, male: “Ah yes, when you get used to them, definitely they will understand that you are solving their problems, they can enlist the clients [sexual partners].”

Especially for the refugee population, health workers explained that extensive counseling is often necessary due to the lack of pre-existing knowledge and awareness about HIV.

Health worker 54-07-2-002, male: “The refugees, most of them are uneducated, so because of that counseling will not be just be an hour or two, it can be like a day.”

*Allowing Time to Process HIV Test Results* Giving index clients time was another approach that was considered beneficial to the listing of sexual partners by index clients.

Health worker 54-04-2-003, male: “But in most cases, there is denial stage. They deny their results. At first, we give them time, we give them time to first decide.”

By planning an ART refill appointment for two weeks after the diagnosis, an opportunity is created to circle back to the topic of APN after the index client has had time to process his or her results.

Health worker 54-07-2-001, male: “[If] someone is not able to list for us [their sexual partners], then we tell him or her to go and think about it. So, when he [or she] comes back, sometimes comes when is a bit relieved, and now can be able to recall and can be able to list for us most of the partners.”

*Employing Counseling Strategies to Promote Sexual Partner Identification* To try to convince index clients to disclose their sexual partners, health workers apply different counseling strategies including emphasizing the benefits of disclosure and testing of sexual partners for the index clients themselves.

Health worker 54-10-2-003, male: “[Counseling a male index client] ‘Now you know your HIV status, will you allow us to disclose to your partner so that she can be responsible in caring [for] you in case you may fall sick? You can have, you can use her to come to be as a treatment supporter, to help you in this process.’”

Health worker 54-04-2-003, male: “[Talking about sexual partner to index client] ‘Maybe that person has the infection but is not taking drug [ART] and is going to infect you more, you will develop an HIV strain that has not been, treatment of which is bad.’”

Other counseling strategies that were mentioned included calling upon the responsibility of the index client to protect others.

Health worker 54-04-2-002, male: “So, we usually tell them, ‘By telling us about your sexual partners, telling us about your sexual life, you are actually saving someone. You are not doing it for yourself alone, you are doing it for your other friends. Maybe that gentleman whom you have just slept with is now talking to your friend, talking to your cousin’. . . . And when you do that, they get a feeling of responsibility, they feel important, they know they now have a what? A role to play in the fight.”

*Collaboration and Partnerships with Those Who Know the Community* To trace sexual partners and convince them to report to the health center for testing, collaboration is sought with third parties such as block leaders (elected leaders in the settlement), village health teams, expert clients (PLHIV who are successfully managing their disease and use their experiences to help others), and health workers from other health centers in those cases where sexual partners have moved to a different region. These people often have intimate knowledge of the local community and can play a role in identifying sexual partners.

Health worker 54-09-2-002, male: “We normally use our expert clients and some of them are able to identify them [sexual partners] by face. . . . we just send the expert client to sight areas of exposed people . . . who are in a relationship with other clients on ART, because they know them.”

Sometimes local leadership helps to design programs to decrease stigma in the community before health workers come to notify sexual partners in that neighborhood.

Health worker 54-02-2-002, female: “We have a list of block leaders in the facility. If we have any outreach, we communicate to them, they mobilize the community and help us sensitize the community.”

*Flexibility in Point of Testing* Health workers narrated that the likelihood of sexual partners reporting for testing is increased by offering to test them at a location of their preference.

Health worker 54-07-2-002, male: “Some we give them appointment, they come to the facility to test willingly. There are some who will say ‘Ah ah for me, I am busy, I am at my workplace, at shop I am selling. If you want to offer for me, come at this time, this is where I am.’”

#### Perspectives on Improving APN

While several strategies are already being employed to facilitate APN, health workers identified a number of areas where the program can be improved and had several recommendations for how the APN program could be made safer and more effective. (Fig. 3, Health worker perspectives on barriers and facilitators of APN and corresponding recommendations to enhance APN safety and effectiveness).

*Enhanced APN Training* Multiple interview participants identified training for health workers as a major area for improving APN. Health workers wanted additional training in counseling strategies, which were considered the strongest factor in whether or not index clients listed their sexual partners. Interview participants also recommended that APN should be implemented at all health center entry points where index clients are identified, and therefore that all health workers should receive APN training, rather than only offering APN training to a small delegation from the ART clinic. Health workers warned that the current lack of awareness about APN among clinicians and other health center staff led to missed opportunities to interview index clients about their sexual partners.

Health worker 54-04-2-002, male: “It is actually better if everyone [all health worker staff] hear about APN. Because it is really needed at every point, it is needed in maternity, it is needed in the IPD [inpatient department]. And then it is not something that has to first go through a clinician or a counselor. The moment you identify someone you can start talking to them and they can tell you everything about their partners, because that's the time when they can open up more, when they have just gotten what? They have known their status.”

One health worker suggested sharing best practices from those health centers that are performing well in APN to amplify best practices. One effective strategy that was mentioned was testing the index client and their sexual partner at the health center simultaneously—as if for the first time—to avoid the discussion of who is to blame for bringing the infection in the relationship. Another effective practice that was described was to set up a community testing site in a neighborhood where a sexual partner of an index client was known to reside, so that the partner could be tested alongside others in the community to avoid stigma.

*Facilitate Transportation of Health Workers* Interview participants recommended addressing the issue of transportation for health workers when performing APN activities, either by providing vehicles such as a motorcycle or a bicycle, or through cash facilitation, making sure to provide this resource before the tracing of sexual partners.

Health worker 54-09-2-001, female: “The APN could be improved because at times we are following these people, at least there should be transport for the healthcare worker to go and follow the partner. And you cannot go alone, it needs at least two or three, because there might be violence or you can be fighting, then the other one can support you. . . . For transport is being facilitated yes, but the distance is also at times far, so the transport [facilitation] might not be enough. . . . In this nearby area it covers, but when you go deep it does not cover.”

*Raising Awareness About the Importance of APN in the Community* Finally, health workers suggested sensitization, or raising awareness about APN and the merits of the program in the general community.

Health worker 54-08-2-002, male: “APN, I think we need more sensitization of people, people should understand, like talk shows and radios what? Programs should be running. . . . People should be told . . . especially for the positive, the importance of this partner notification. Or sometimes if like here, [there are] issues of culture that could bury people from disclosing. Or you find another person is negative, the other one is negative what it means. People should be sensitized about these services, so that I, if I hear on the radio, [and] maybe tomorrow somebody calls me and notifies me [notification of possible HIV exposure] I know this could be ABCD [that this could be the APN program], people would be able to respond.”

Sensitization was thought to hold potential especially for specific refugee tribes from South Sudan like the Dinka and Nuer tribes who had been mentioned by health workers in relation to a number of incidents of disclosure-related violence in this setting. Health workers reported these tribes were said to have lived in relative isolation in South Sudan and therefore had had little exposure to HIV awareness interventions in the past. A solution they proposed was targeted sensitization activities working closely with community leaders to promote the acceptance of HIV and HIV testing and diagnosis, and thereby improve the safety of APN.

Health worker 54-01-2-003, male: “I would think about community sensitization and community dialogue. You know, for one good thing in refugee settings, they hear and understand their elders better than any other person. . . . anything that comes from their opinion leaders, they take from there. So, when we involve their elders, the church leaders, the opinion leaders . . . telling them that HIV is not, is not one man's disease—it cuts across. It does not select whether you are Ugandan, you are Sudanese, you are an American, you are an Indian, does not select. It does not select, so all can be affected by HIV. So, one is community dialogue that would help so much.”

#### The Necessity of Optimizing APN for Refugees

In interviews with health workers, several refugee-specific factors were reported that put refugees at high risk of HIV exposure highlighting the importance of optimizing APN for this population so that these behaviorally vulnerable individuals can be identified and tested. Health workers described that many refugees may have been exposed to sexual violence while fleeing to Uganda, and also that cultural practices of specific refugee tribes, including widow inheritance and polygamy may facilitate HIV transmission.

Health worker 54-07-2-01, male: “Them being refugees, they are coming in . . . some of them were raped on the way, and some of them, we don’t know who raped them. And sometimes a woman [who] has been raped, fears to disclose to the husband because maybe the husband might chuck the woman. . . . They think maybe the husband will divorce her since she has been penetrated by unknown people.”

Health worker 54-03-2-003, female: “The refugees . . . their culture, when your husband is maybe in the army, [you are] remaining behind. [His] brothers who are here, can take control of you in everything. So, [the woman] can even go ahead go having the sexual intercourse with the brother, with the in-laws, without even knowing the status. . . . And you find one [man] maybe may even have like six women, twelve, eleven there easily.”

Health worker 54-07-2-001, male: “[Refugees], polygamy is part of them . . . part of their culture. So, you find . . . this widow inheritance . . . that means . . . . a man takes over a woman of the brother, maybe that older brother has died . . . sometimes you find that like these refugees they are . . . four boys in the home, we are all married . . . three of us they are soldiers so . . . they trust me [the fourth brother] to escort their wives to Uganda as refugees, but for them they remain their side fighting. So, you find that me who has been entrusted, I end up start using these women because their husbands are not around.”

The presence of money and humanitarian aid makes refugee settlements attractive sites for trade and business, and transactional sex is common in this setting.

Health worker 54-06-2-003, male: “This is a refugee setting area base camp . . . [there is a] small trading center here, because there are different tribes . . . because work . . . [is] bringing many people here, there is money. . . . Even sex workers we have here, since . . . just men workers are here, they are getting money, so you find now mostly people here . . . in night working two to three partners, making money.”

Interview participants explained that the refugee settlements are inhabited for the large part by women and children. Many of the husbands of these refugee women stayed behind in their home country to work or fight in the war. Family planning and HIV prevention are delicate subjects to discuss with these women as the societal expectation is that due to the absence of their husbands these measures are not necessary.

Health worker 54-07-2-002, male: “Our clients from the refugee side they are female . . . the reason being is most of the women are here. Some their husbands have died, some their husband has run to central Africa . . . so mostly the camp has been constituted by women. Find also that the women . . . someone who is married and has been staying with a man, to stay alone is always very hard. So, there are more chances they have been exposed to HIV.”

Health worker 54-06-2-001, female: “It is more hard to get someone married and you ask them if they had unprotected sex outside . . . Some are positive, the husband [is] in Sudan. When they come here and we are telling them to start on family planning here, [they say] ‘but my husband is in Sudan’ and you don't want to say, ‘but you are not limited to your husband’.”

### Convergent Mixed Methods Findings

In examining the interview transcripts and APN register data together, health workers’ impression that APN is an effective program was concordant with the high yield of new HIV diagnoses obtained through APN-based targeted testing (a yield much higher than the population prevalence in West Nile). Interview participants’ observation that the APN program could reach groups with lower health-seeking behavior such as men was corroborated by the equal representation of men and women among index clients and sexual partners. The low average number of sexual partners per index client documented in APN registers emphasized the challenges described by health workers in convincing index clients to identify their sexual partners. While the median number of sexual partners identified per index client was the same for both refugees and Ugandan nationals, the wider IQR for Ugandan nationals and statistically significant Mann–Whitney U test suggest that Ugandan nationals report more sexual partners than refugees. Given the concerns health workers express in qualitative interviews about underreporting of sexual partners by refugees and the multiple valid reasons they describe why refugees may be hesitant to disclose sexual partners including fear of violence and abandonment, it is possible that the number of sexual partners listed per index client is falsely low for refugees. Both quantitative and qualitative findings identified *assisted notification* to be the most common and most effective notification option, with both sets of findings highlighting the high percentage of *self-notification* cases in which assistance by a health worker is later employed. The divergence in distribution of notification option choice that was seen between health centers may be explained by the lack of consensus among health workers regarding the definitions of the three notification options described in the qualitative interviews. While interview participants described challenges in notifying sexual partners and convincing sexual partners to report to health center facilities for testing, APN register data reveal that the majority of notified partners reported to the health center and agreed to be tested.

## Discussion

Since the initiation of APN services at the health centers in West Nile included in this study, over 880 index clients participated in APN and more than 1100 sexual partners were identified, which is an average sexual partner to index client ratio comparable to that found in other APN studies in Uganda [51, 52]. As a result of APN services, 139 people have been newly diagnosed with HIV, providing them with the opportunity to link to care and prevent further disease transmission in the community.

While the yield of 14% new HIV diagnoses found in this study appears low compared to the 29% yield found in the 2016 Ugandan pilot study that motivated the national expansion of APN services [25], it is comparable to the most recent MoH data on APN implementation in Uganda. APN cascade data from 705 of Uganda’s 2500 health centers reveal that between January and September 2018, testing of 22,663 sexual partners of index clients led to 9211 HIV diagnoses (HIV prevalence of 27%), of which 4561 were sexual partners not enrolled in care (new diagnoses yield of 14%) [53]. Studies on APN, including a number of large randomized-controlled trials in SSA [22, 24, 54], consistently show partner notification services lead to higher uptake of HIV testing and result in the diagnosis of high proportions of sexual partners [10]. Interviews conducted with health workers in West Nile in this study support this assertion and demonstrate widespread consensus about the merits of the APN program and its potential to reach behaviorally vulnerable groups.

While the APN program is effective in yielding new HIV diagnoses, findings from the current study suggest that several barriers to APN persist and that characteristics of the West Nile refugee settlement context pose unique challenges to APN implementation. In West Nile, HIV continues to carry connotations of promiscuous behavior and a bad prognosis. Index clients are fearful to share their status due to fear of violence, stigmatization and termination of relationships that may follow HIV status disclosure. The interconnectedness of stigma, IPV and HIV status disclosure has been well-documented and fear of potentially negative consequences inhibits disclosure in other SSA care settings [55–57]. In West Nile, fear not only influences the identification of sexual partners, but all facets of APN, contributing to the choice of less effective notification options, leading to delays in disclosure when *self-notification* is selected, and preventing sexual partners from testing for HIV.

In this context, APN becomes a highly time- and labor-intensive strategy. The APN process is described as a lengthy dance between health worker and index client, in which a spectrum of counseling strategies is deployed to build the foundation of friendship and trust needed for index clients to feel comfortable disclosing the details of their sexual partners. Once listed, the process of tracing these sexual partners requires substantial human and material resources. As a result of the high degree of mobility of refugees, health workers frequently have to travel beyond settlement boundaries and sometimes beyond region borders to trace sexual partners. These efforts have to be undertaken after the completion of regular clinic duties or on weekends and frequently require upfront costs be covered by the health workers themselves.

Findings from the current study demonstrate that refugee settlements are a unique context and that refugees represent a distinct population that makes significantly different APN choices compared to Ugandan nationals. Fear of negative consequences such as violence and abandonment likely leads to underreporting of sexual partners by refugee index clients, which is cause for concern as interviews with health workers reveal refugee-specific behavioral characteristics that put this population at risk for HIV exposure. In West Nile, compared to Ugandan nationals, refugees have been shown to choose *self-notification* less commonly, a less effective modality to bring sexual partners to health centers for testing. A possible explanation for this finding may be the low feasibility of personally disclosing to sexual partners for refugees. Mobility among the refugee population is high and it is not uncommon for sexual partners to be located far away. Other possible contributing factors alluded to by interview participants are the cultural customs and norms surrounding marriage and acceptable sexual behaviour held by specific refugee tribes who have had low exposure to HIV awareness activities making *self-notification* particularly challenging.

Of the different challenges, the lack of willingness of index clients to list their sexual partners was considered to be the main barrier to APN effectiveness in the West Nile refugee settlement. While logistical and financial difficulties are associated with sexual partner tracing, the high percentage of tested sexual partners for each notification option (87%, 96% and 91%, for *self-notification*, *assisted notification* and *provider notification*, respectively) demonstrates that only a minority of sexual partners is never reached or refuses to be tested. These findings are at odds with other studies on APN which have found a much lower rate of sexual partner testing and more apparent differences in referral rates between the respective notification options [19, 22, 24]. The high testing rates observed in West Nile are a testament to the (perhaps unsustainable) time and resources invested by health workers to trace sexual partners–time and resources often diverted away from other clinic activities or personal time. The greatest potential for APN optimization and prevention of further HIV transmission in this setting lies in increasing the proportion of sexual partners that index clients identify by addressing the fear of disclosure-related violence and stigmatization that index clients experience. To do this, further research is needed that gives insight into the extent to which negative consequences such as violence influence and result from APN participation.

The findings of this study should be considered in the context of the study’s limitations. The varying definitions used by health workers of the different notification options, combined with the high proportion of *self-notification* cases that subsequently received health worker-mediated forms of notification draws into question the conclusions that can be drawn regarding the associations between notification option choice and various outcomes. The barriers and facilitators for index clients examined in this study apply only to those index clients who agreed to take part in the APN program. The number of index clients who are offered APN that declines participation is not routinely recorded. The low uptake found in other APN studies from Uganda and SSA [51, 52, 58] suggests that this proportion may be significant. This group may experience a unique spectrum of barriers that remained uninvestigated in this study. While health workers play an important role in APN and their views and experiences offer valuable insight into the APN process, the perspective of index clients and their sexual partners—the people who do and do not participate in APN and who experience the potential negative consequences associated with disclosure through APN—have not been examined in this study. Finally, study findings apply to a limited area of the West Nile region. Cultural practices and population composition differ significantly within and between regions highlighting that the perspectives represented in this study may not be generalizable to all settings.

## Conclusion

Quantitative data from APN and HIV registers and interviews with health workers involved in APN in refugee settlements in West Nile Uganda demonstrate that while APN effectively identifies individuals at a high-risk for HIV and leads to new HIV diagnoses, context-specific barriers to APN exist in refugee settlements. While logistical and practical challenges hamper implementation of APN in this setting, the greatest potential for APN optimization lies in addressing HIV-related stigma to attenuate the fear of negative consequences associated with HIV status disclosure. Health education and sensitization can play an important role in this regard. Prospective research involving index clients and sexual partners is needed that explores how APN participation is shaped by fear of negative consequences and investigates whether APN is associated with incidents of interpersonal violence in refugee settlements to optimize APN in this context.
